# Biological Effects of Bioactive Glass-Containing Self-Adhesive Resin Cements on Dental Pulp Stem Cells

**DOI:** 10.3390/jfb17050215

**Published:** 2026-05-01

**Authors:** Jiyoung Kwon, Seung Woo Chae, Hyun-Jung Kim

**Affiliations:** 1 Department of Conservative Dentistry, Kyung Hee University Dental Hospital, Seoul 02447, Republic of Korea; jykt55@gmail.com; 2 Department of Conservative Dentistry, School of Dentistry, Kyung Hee University, Seoul 02453, Republic of Korea; chaesw517@naver.com

**Keywords:** bioactive glass, self-adhesive resin cement, dental pulp stem cell

## Abstract

The aim of this study was to evaluate the biological effects of bioactive glass-containing self-adhesive resin cements (SARCs) on human dental pulp stem cells (DPSCs), focusing on cytocompatibility, odontogenic differentiation, and mineralization. Experimental SARCs containing 0–5 wt% BAG (BG0–BG5) were compared with two commercially available SARCs, RelyX U200 and TheraCem. Eluates were prepared and applied to DPSCs for the methylthiazol tetrazolium (MTT) assay, quantitative real-time polymerase chain reaction (qRT-PCR), immunofluorescence (IF) staining, and Alizarin Red S (ARS) staining. The result showed there were no significant differences in cell viability across all groups (*p* > 0.05), indicating that the addition of BAG did not affect cell viability, while the early odontogenic differentiation markers, such as *RUNX2*, *ALP*, and *COL1A1*, showed no clear trend among the groups. However, late-stage markers (*DMP-1* and *DSPP*) were significantly higher in the BG2–BG5 groups relative to the OM group (*p* < 0.05). IF staining revealed intense signals in the BG2–BG5 groups (*p* < 0.05) and also ARS staining showed a time-dependent increase in mineral deposition. Within the limitations of this study, BAG-containing SARCs do not negatively impact cytocompatibility and promote late-stage odontogenic differentiation and mineral deposition.

## 1. Introduction

As adhesive restorative procedures become increasingly complex, the risk of procedural failure and complications related to technique sensitivity correspondingly rises. To address these limitations, self-adhesive resin cements (SARCs) have been introduced into the dental market as simplified luting materials. By eliminating the multiple steps inherent to conventional adhesive cementation protocols, such as acid etching, priming, and bonding, SARCs streamline the cementation process and facilitate more efficient clinical applications. Owing to their simplified handling and reduced technique sensitivity, SARCs have been widely adopted across a broad spectrum of indirect restorative procedures.

Nevertheless, because SARCs intrinsically depend on directly modifying the tooth substrate to obviate separate pretreatment steps, their potential to induce pulpal irritation must be carefully considered, particularly in clinical situations involving thin residual dentin [1]. This concern is especially relevant in deep cavity preparations or minimally invasive indirect restorations, where the pulp–dentin complex may remain highly vulnerable to chemical irritation. In such cases, the leachable acidic monomers and residual components may diffuse through dentinal tubules and adversely affect the underlying pulp tissue [2,3]. Although dentin serves as a partial diffusion barrier, its protective capacity strongly depends on the thickness of the remaining dentin, and toxic components may more readily reach the pulp when the dentin is limited. When applied directly to dentin surfaces, SARCs may release acidic species during polymerization, thus locally reducing the pH. These acidic byproducts can penetrate dentinal tubules, thereby altering the pulpal microenvironment and potentially triggering inflammatory or cytotoxic responses [2]. Furthermore, unpolymerized resin monomers, including 2-hydroxyethyl methacrylate (HEMA) and triethylene glycol dimethacrylate (TEGDMA), may infiltrate the dentin and interact with cells within the pulp–dentin complex [4,5]. Such monomers have been associated with oxidative stress, mitochondrial dysfunction, and reduced cellular viability [6,7]. Collectively, these interactions may compromise not only cell survival but also the odontogenic differentiation and mineralization capacity of dental pulp stem cells (DPSCs), ultimately impairing the regenerative potential of pulp tissue by disrupting the expression of key odontogenic markers, such as alkaline phosphatase (*ALP*), runt-related transcription factor 2 (*RUNX2*), dentin sialophosphoprotein (*DSPP*), and dentin matrix protein 1 (*DMP-1*).

DPSCs play a pivotal role in pulpal repair and regeneration and contribute to tertiary dentin formation and odontoblast-like cell differentiation in response to injury [8,9,10]. Accordingly, disruption of DPSC viability or function may compromise dentin–pulp complex homeostasis and jeopardize long-term pulpal vitality [11,12]. In this context, improving the biological compatibility of SARCs with DPSCs is of particular clinical importance. Strategies for minimizing the release of cytotoxic components [13], regulating material-associated acidity [14], and incorporating bioactive functionalities [15] may contribute to preserving pulpal health while supporting dentin regeneration. Given that restorative materials can directly influence DPSC behavior, the biological effects of SARCs on dental pulp are closely linked to the long-term success and durability of restorative outcomes. Consequently, the development of SARCs with enhanced biocompatibility and favorable interactions with DPSCs is critical for achieving predictable and biologically sound restorations. Importantly, the cytotoxicity of resin-based restorative materials is not static and may evolve over time, as the release of residual monomers and acidic byproducts tends to decrease with aging [16]. However, the long-term biological behavior of SARCs remains poorly understood, highlighting the need to be evaluated under both immediate and aged conditions.

To overcome these intrinsic biological limitations, bioactive glass (BAG) has emerged as a promising functional additive with significant therapeutic potential. Originally developed by Larry Hench in 1969, BAG was designed to have excellent biocompatibility and a unique ability to bond directly with both hard and soft tissues without eliciting adverse biological responses [17]. Upon exposure to aqueous physiological environments, BAG undergoes a series of surface reactions characterized by the release of biologically active ions, such as calcium and silicate, which contribute to buffering acidic microenvironments. This ionic exchange initiates the formation of a silica-rich layer, followed by the precipitation and maturation of calcium phosphate phases, ultimately resulting in the development of a hydroxycarbonate apatite layer that closely resembles the mineral phase of natural hard tissues [17,18].

In addition to its physicochemical interactions, BAG promotes cellular adhesion, proliferation, and differentiation by modulating the local microenvironment through sustained ion release [19,20,21]. BAG-derived ionic dissolution products have also been reported to upregulate odontogenic differentiation pathways and modulate inflammatory signaling, suggesting their potential therapeutic benefits for pulp tissue preservation [22,23]. Through these combined effects, BAG may establish a biologically favorable milieu that supports mineralization and potentially induces odontogenic differentiation by regulating cellular behavior and extracellular matrix formation.

Substantial research has been directed toward incorporating BAG into dental restorative materials. However, most previous investigations have primarily focused on improving the physicochemical and mechanical performance, whereas the biological implications of BAG-modified restorative materials remain comparatively underexplored. Yun et al. [24] demonstrated that incorporating BAG into dental sealants significantly increased enamel microhardness and the shear bond strength. Similarly, Kim et al. [25] observed that BAG-modified dentin adhesives reduced dentin permeability and improved bond strength by promoting interfacial remineralization. In addition, Jang et al. [26] showed that composite resins supplemented with BAG could induce dentin surface remineralization while increasing microhardness.

Collectively, these findings highlight the promising functional benefits of incorporating BAG into restorative materials, particularly with respect to the enhancement of remineralization and mechanical stability. Nevertheless, the biological influence of SARC formulations may be especially relevant, given the intrinsic acidity and direct dentin contact characteristics of SARCs, which raise concerns regarding pulpal compatibility. For successful incorporation of BAG into SARCs, determining the optimal concentration is crucial. Previous findings showed that a concentration of BAG below 1 wt% was insufficient bioactivity for effective remineralization, while exceeding 5 wt% was risky because it compromised mechanical stability via water sorption and may induce cytotoxicity [19,27]. Therefore, BAG-containing SARCs should be further investigated, particularly with respect to their interactions with pulp-derived stem cells within this optimized concentration range.

Accordingly, this study aimed to evaluate the biological effects of experimental SARCs containing 0, 1, 2, 3, 4, and 5 wt% BAG on human DPSCs, with particular emphasis on cell viability, odontogenic differentiation, and mineralization potential. The null hypotheses tested were as follows: (1) eluates from SARCs would not affect the viability of DPSCs, (2) inflammatory gene expression and mineralization capacity would not differ among materials, and (3) the biological responses induced by SARCs would not significantly change over time.

## 2. Materials and Methods

### 2.1. Materials

The experimental SARCs were formulated and supplied by Mediclus (Cheongju, Republic of Korea). The BAG particles were incorporated into the SARCs at concentrations of 0, 1, 2, 3, 4, and 5 wt%, which are hereafter designated as BG0 through BG5. Two commercially available SARCs, RelyX U200 (RU; 3M ESPE, St. Paul, MN, USA) and TheraCem (TC; Bisco, Schaumburg, IL, USA), served as controls. The compositions of all the investigated materials are detailed in Table 1.

The eluates of the resin cements were prepared according to the ISO 10993-12 standard guidelines [28]. Disk-shaped specimens (5 mm in diameter and 2 mm in thickness) were prepared and light-cured for 40 s according to the manufacturer’s instructions. To satisfy the surface area-to-volume ratio of 3 cm^2^/mL as specified by the ISO standard, the total surface area of each specimen (approximately 0.707 cm^2^) was calculated, and the appropriate volume of either growth medium (GM) or odontogenic medium (OM) was deter-mined. The specimens were then immersed in the medium and incubated at 37 °C in a humidified atmosphere of 5% CO_2_ for 24 h.

The compositions of GM and OM are detailed in Table 2. Following the extraction period, all media were sterilized via filtration through 0.2 μm syringe filters (Pall Life Sciences, Port Washington, NY, USA) and utilized immediately for biological assays. Human DPSCs (CefoBio, Seoul, Republic of Korea) were cultured in GM and maintained at 37 °C in a humidified atmosphere containing 5% CO_2_. Cells from the fifth passage were used in all the experiments to ensure methodological consistency.

**Figure 1:**
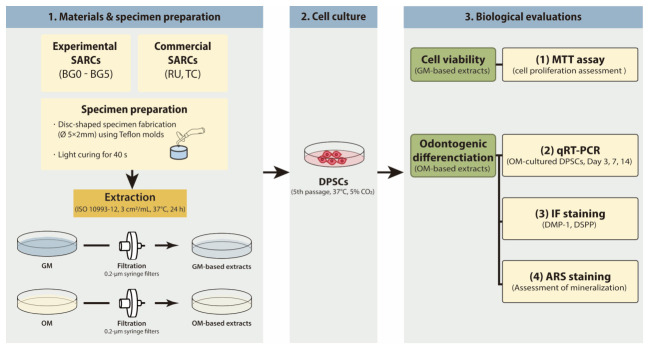
Experimental flow chart. RU, Rely X U200; TC, TheraCem; GM, growth medium; OM, odontogenic medium; DPSCs, dental pulp stem cells; self-adhesive resin cement, SARC; MTT, methylthiazol tetrazolium; qRT-PCR, quantitative real-time polymerase chain reaction; IF, immunofluorescence; ARS, Alizarin Red S.

GM-based extracts were employed for the methylthiazol tetrazolium (MTT) assay to evaluate cell viability, whereas OM-based extracts were used for quantitative real-time polymerase chain reaction (qRT-PCR), immunofluorescence (IF) staining, and Alizarin Red S (ARS) staining to assess odontogenic differentiation potential. The experimental workflow is schematically illustrated in Figure 1.

### 2.2. MTT Assay

To assess cell proliferation, DPSCs were seeded in 96-well plates (SPL, Pocheon-si, Gyeonggi-do, Republic of Korea) at a density of 2 × 10^4^ cells/mL in 100 μL of GM and incubated for 24 h. Subsequently, the medium was replaced with 200 μL of eluate at dilution ratios of 1:2 and 1:4. The MTT assay (Sigma-Aldrich, St. Louis, MO, USA) was performed by adding 20 μL of MTT solution (5 mg/mL) to each well, followed by a 4 h incubation at 37 °C. The supernatant was then removed, and the resultant formazan crystals were dissolved in 100 μL of dimethyl sulfoxide (Sigma-Aldrich). After 30 min of agitation on a microplate shaker, the absorbance at 570 nm was measured using a microplate reader (Spark 10M, Tecan, Männedorf, Switzerland).

### 2.3. qRT-PCR

The expression of genes associated with odontogenic differentiation was quantified via qRT-PCR. Total RNA was isolated from DPSCs cultured in OM for 3, 7, and 14 days using the TRIzol reagent (Molecular Research Center Inc., Cincinnati, OH, USA) according to the manufacturer’s protocol. One microgram of total RNA was reverse-transcribed into cDNA using the PrimScript^TM^ First Strand cDNA Synthesis Kit (Takara Korea Biomedical Inc., Seoul, Republic of Korea).

Amplification was conducted using qPCRBIO SyGreen Mix Hi-ROX (PCR Biosystems, London, UK) on a StepOnePlus^TM^ Real-Time PCR system (Applied Biosystems; Thermo Fisher Scientific, Waltham, MA, USA). The expression levels of early markers (*RUNX2*, *ALP*, and collagen type I alpha 1 (*COL1A1*)) and late markers (*DMP-1* and *DSPP*) were analyzed. The primer sequences for the target genes are listed in Table 3. Expression levels were normalized to β-actin and calculated using the 2-ΔΔCT Method, with results reported as fold changes relative to the control group [29].

### 2.4. IF Staining

For the IF detection of *DMP-1* and *DSPP* expression, DPSCs were seeded at a density of 2000 cells/well onto sterilized coverslips in eight-well chamber slides. On day 7, the cells were rinsed three times with phosphate-buffered saline (PBS) and fixed with 4% paraformaldehyde (Sigma-Aldrich) for 30 min at room temperature. Following permeabilization with 0.1% Triton X-100 and inhibition of endogenous peroxidase activity with 3% hydrogen peroxide for 20 min, non-specific binding was blocked using 0.5% goat serum in PBS.

Samples were incubated overnight at 4 °C with rabbit anti-human *DMP-1* and *DSPP* primary antibodies (1:100; Santa Cruz Biotechnology Inc., Dallas, TX, USA). Subsequently, cells were incubated with Alexa Fluor 488-conjugated goat anti-mouse secondary antibody (1:1000; Invitrogen, Carlsbad, CA, USA) for 1 h at 37 °C. Nuclei were counterstained with 4′,6-diamidino-2-phenylindole (DAPI; Sigma-Aldrich), and images were captured using a confocal microscope (LSM700; Carl Zeiss, Jena, Germany). The fluorescence intensity was quantified as the mean gray value using ImageJ software (version 1.53t; NIH, Bethesda, MD, USA) and expressed as a relative fluorescence intensity.

### 2.5. ARS Staining

To evaluate mineralized nodule formation, DPSCs were seeded in 12-well plates at 2 × 10^4^ cells/well. The positive control group was maintained in OM to induce differentiation, while the negative control was maintained in GM. The cultures were incubated for up to 28 days, and the medium was replaced every 48 h. On days 14, 21, and 28, the cells were washed with PBS and stained with ARS. For quantification, the stain was solubilized using 10% cetylpyridinium chloride, and the optical density (O.D.) was measured at 560 nm using a SpectraMax ELISA reader (Molecular Devices, San Jose, CA, USA).

### 2.6. Statistical Analysis

All experiments, including the MTT assay, qRT-PCR, and ARS staining, were performed in triplicate (*n* = 3) to ensure the reproducibility of the results. For multiple comparisons across groups, one-way analysis of variance (ANOVA) was conducted, followed by Tukey’s post hoc test to identify specific group differences in the MTT assay, qRT-PCR, IF staining intensity, and relative O.D. in the ARS staining assay. Statistical analyses were performed using GraphPad Prism 10.6.1 (GraphPad Software Inc., San Diego, CA, USA).

## 3. Results

### 3.1. Cell Viability (MTT Assay)

**Figure 2:**
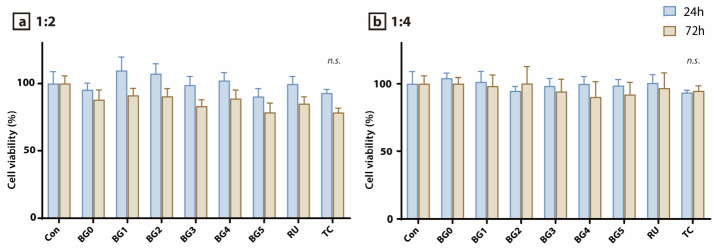
MTT-assay-based evaluation of cell viability in human DPSCs. Cell viability was assessed under two dilution conditions: (**a**) 1:2 and (**b**) 1:4. Measurements were performed at 24 and 72 h. No statistically significant differences in cell viability were observed among the experimental groups for either dilution condition (*p* > 0.05). n.s. indicates no significant difference among the groups (*p* > 0.05).

The viability of DPSCs following exposure to eluates from the experimental SARC specimens at dilution ratios of 1:2 and 1:4 is presented in Figure 2a and Figure 2b, respectively. No statistically significant differences were observed among the experimental groups at either 24 or 72 h (*p* > 0.05). Under both dilution conditions, the cell viability at 72 h decreased slightly relative to that at 24 h. Furthermore, no significant differences in cell viability were detected between 1:2 and 1:4 dilution ratios at either time point.

### 3.2. Gene Expression Analysis (qRT-PCR)

**Figure 3:**
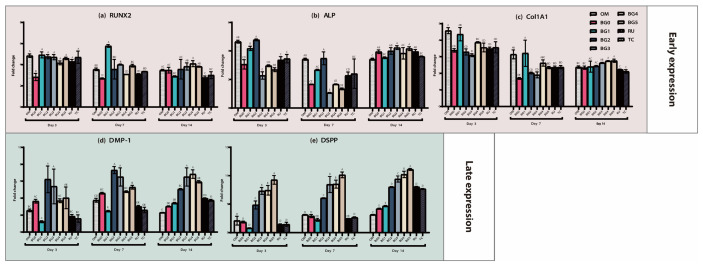
qRT-PCR analysis of odontogenic-differentiation-related gene expression. The expression levels of early odontogenic differentiation markers—(**a**) *RUNX2*, (**b**) ALP, and (**c**) *COL1A1*—and late odontogenic differentiation markers—(**d**) *DMP-1* and (**e**) *DSPP*—were analyzed by qRT-PCR. Gene expression was evaluated at days 3, 7, and 14. No consistent trends were observed in the expression of early-stage odontogenic markers among the experimental groups. In contrast, BG2–BG5 showed a more significant upregulation of the late-stage odontogenic markers *DMP-1* and *DSPP* compared with the OM group (*p* < 0.05). Different uppercase letters on the bars indicate statistically significant differences among the group (*p* < 0.05).

The mRNA expression levels of the odontogenic differentiation markers in this study, *RUNX2*, ALP, and *COL1A1*, are shown in Figure 3a–c. *RUNX2* expression was significantly higher in the BG1 group than in the OM group at day 7 (*p* < 0.05), whereas all other groups exhibited expression levels comparable to or lower than those of the OM group across all time points (Figure 3a). For *ALP*, the BG0, BG2, BG3, BG5, and RU groups showed significantly elevated expression compared with the OM group on day 14 (*p* < 0.05), whereas the remaining groups demonstrated comparable or lower expression at all evaluated time points (Figure 3b). *COL1A1* expression was significantly increased in the BG4 and BG5 groups relative to that in the OM group at day 14 (*p* < 0.05); however, no other group showed significant upregulation at any time point (Figure 3c). Overall, no consistent or dose-dependent trend was observed among the early odontogenic differentiation markers, suggesting that BAG incorporation had a limited effect on early-stage differentiation.

The mRNA expression levels of *DMP-1* are shown in Figure 3d. On day 3, the BG2 and BG3 groups exhibited significantly higher expression than the OM group (*p* < 0.05). On day 7, expression was significantly higher in the BG2, BG3, and BG5 groups than that in the OM group, whereas the BG1 group showed significantly lower expression (*p* < 0.05). By day 14, the BG2–BG5, RU, and TC groups showed significantly higher *DMP-1* expression levels than the OM group (*p* < 0.05).

The *DSPP* mRNA expression levels are shown in Figure 3e. At days 3 and 7, *DSPP* expression was significantly upregulated in the BG2–BG5 groups compared with the OM, BG0, BG1, RU, and TC groups, with the highest expression observed in BG5. Among these, the BG5 group showed the highest expression levels, followed by the BG4 and BG3 groups, all of which were significantly higher than those observed in the BG2, RU, and TC groups (*p* < 0.05).

### 3.3. Immunofluorescence Analysis (IF Staining)

**Figure 4:**
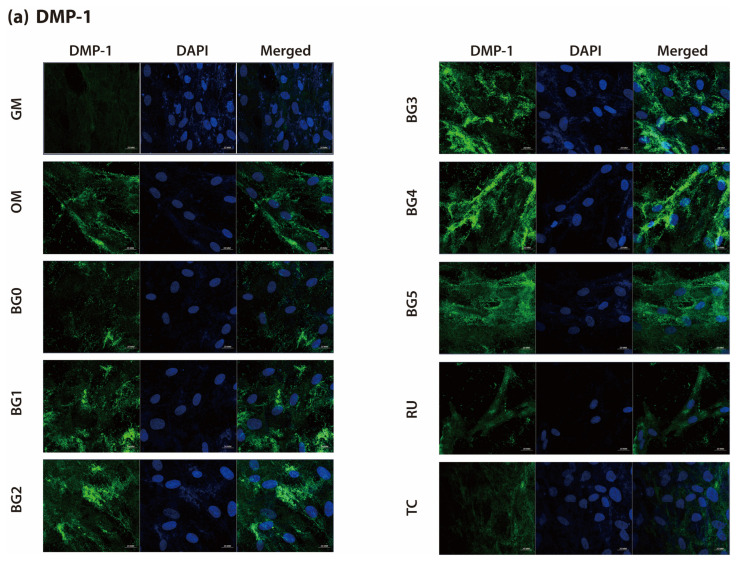

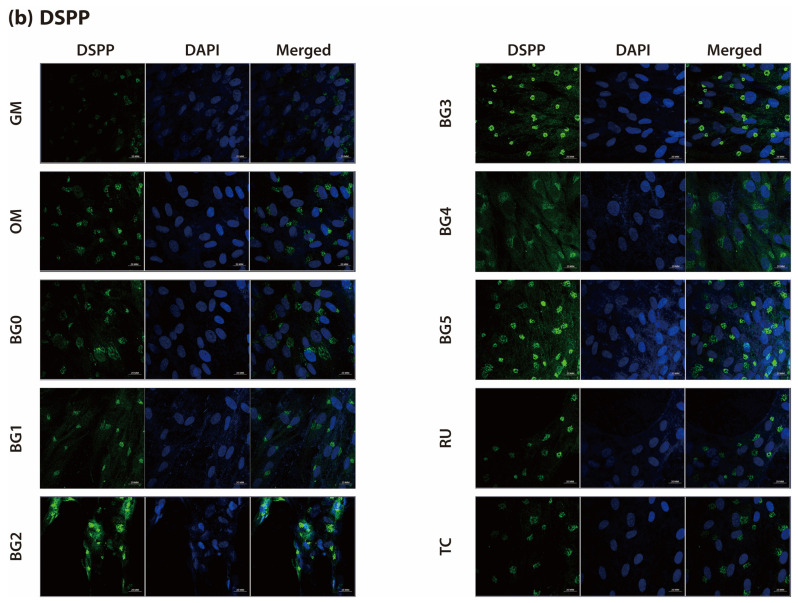
Immunofluorescence (IF) staining images showing *DMP-1* (**a**) and *DSPP* (**b**) expression on day 7. The BG2–BG5 groups demonstrated noticeably stronger fluorescence than the OM group, which exhibited moderate signals, whereas the GM group showed minimal fluorescence.

IF staining results for *DMP-1* and *DSPP* are shown in Figure 4a and Figure 4b, respectively. The fluorescence intensity distinctly differed between experimental groups. The GM group exhibited minimal fluorescence signals, whereas those of the OM group were moderate. In contrast, the BG1–BG5 groups demonstrated stronger fluorescence signals than the OM group, with notably intense staining observed in the BG2, BG3, BG4, and BG5 groups.

**Figure 5:**
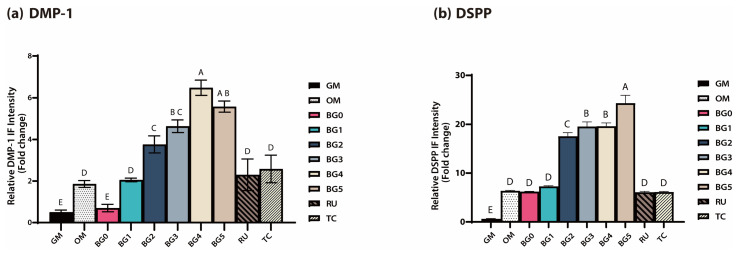
Quantitative analysis of *DMP-1* (**a**) and *DSPP* (**b**) fluorescence intensity obtained from IF staining images using ImageJ software. BG2–BG5 groups exhibited significantly higher relative fluorescence intensities than the OM group (*p* < 0.05), whereas RU and TC showed levels comparable to OM. Different uppercase letters on the bars indicate statistically significant differences among the group (*p* < 0.05).

The fluorescence intensity was quantitatively analyzed using ImageJ software, and the results are presented in Figure 5a,b. The relative fluorescence intensities of *DMP-1* and *DSPP* in the BG2–BG5 groups were significantly higher than those observed in the OM group (*p* < 0.05), whereas those of the RU and TC groups were comparable to those of OM. Among the BG groups, BG4 showed the highest fluorescence intensity for *DMP-1*, whereas BG5 exhibited the highest fluorescence intensity for *DSPP* (*p* < 0.05).

### 3.4. Mineralized Nodule Formation (ARS Staining)

**Figure 6:**
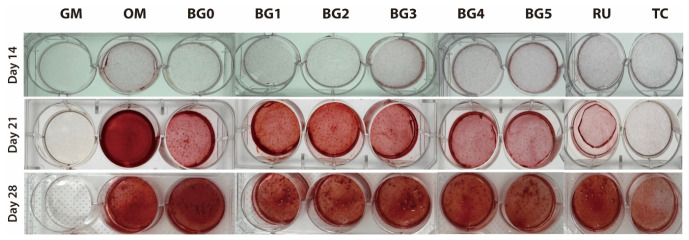
Representative images of ARS staining. ARS staining showed progressive mineral deposition on days 14, 21, and 28. OM showed intensive coloration, whereas GM and TC exhibited minimal or no staining. The BG groups showed visible red staining from day 21 onward, which intensified by day 28.

As shown in Figure 6, ARS staining demonstrated a time-dependent increase in mineral deposition. Minimal or no red staining was observed in the GM and TC groups, whereas the OM group exhibited intense red coloration, confirming its role as a positive control. In contrast, the BG groups showed detectable red staining from day 21 onward, with markedly stronger staining observed in the BG1–BG5 groups at day 28, indicating more extracellular matrix mineralization compared with that in the GM and TC groups.

**Figure 7:**
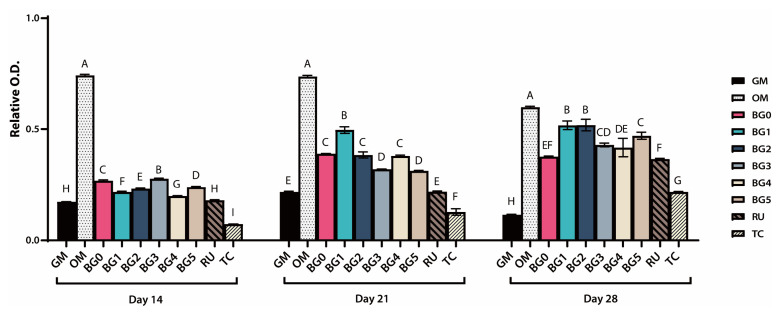
Quantitative analysis of ARS staining. The relative optical density (O.D.) demonstrated a progressive increase in mineralization over time. OM consistently exhibited the highest values, while BG1 and BG2 showed a higher O.D. than the other groups. TC showed the lowest values. (*p* < 0.05). Different uppercase letters on the bars indicate statistically significant differences among the group (*p* < 0.05).

Consistent with these qualitative findings, the quantitative analysis of the relative O.D. presented in Figure 7 reveals a progressive increase in mineralization from days 14 to 28. The OM group exhibited the highest O.D. values at all time points (*p* < 0.05). On day 14, most BG groups showed comparable O.D. values, whereas the TC group demonstrated significantly lower values than the GM group (*p* < 0.05). On day 21, the BG1 group exhibited significantly higher O.D. values than the RU and TC groups (*p* > 0.05), and the BG groups collectively showed significantly higher O.D. values than the GM, RU, and TC groups (*p* < 0.05). On day 28, the BG1 and BG2 groups demonstrated significantly higher O.D. values than the other BG groups and the RU and TC groups. However, these values were significantly lower than those in the OM group (*p* < 0.05). The TC group consistently exhibited the lowest O.D. values at all time points (*p* < 0.05).

## 4. Discussion

This study evaluated the biological effects of BAG-containing SARCs on DPSCs, particularly their viability, odontogenic differentiation, and mineralization potential. Overall, exposure to eluates from the experimental formulations did not significantly compromise DPSC viability under either dilution condition at 24 or 72 h. Thus, the first null hypothesis, that SARCs would not affect DPSC viability, was accepted. In contrast, BAG-containing groups demonstrated more pronounced odontogenic differentiation-related responses than the controls, as evidenced by the consistent upregulation of the late-stage markers *DMP-1* and *DSPP* at the transcript level and their corresponding increases in IF intensity, particularly in BG2–BG5. Moreover, BAG-modified formulations promoted extracellular matrix mineralization over time, with detectable mineral deposition emerging from day 21 and becoming more evident by day 28. Accordingly, the second null hypothesis that no differences would be observed in the differentiation or mineralization capacity among the materials was rejected. In addition, the observed time-dependent increase in mineralization suggests that the biological responses were not entirely stable throughout the experimental period, leading to the rejection of the third null hypothesis. Collectively, these findings indicate that although BAG incorporation did not adversely affect short-term cytocompatibility, it may provide a microenvironment more favorable to differentiation and mineralization for DPSCs, supporting the potential of BAG-containing SARCs as biologically improved materials.

The MTT assay results showed that exposure of DPSCs to eluates from the experimental BAG-containing SARCs did not significantly compromise cell viability under either dilution conditions (1:2 or 1:4) at either 24 or 72 h. No significant differences were observed among the experimental groups, indicating that BAG incorporation of up to 5 wt% did not induce overt short-term cytotoxicity (Figure 2). Importantly, the cell viability values in the BAG-modified formulations were comparable to those of the commercially established control cements, RU and TC, which were selected as reference materials because of their widespread clinical use and frequent inclusion as benchmark SARCs in previous biological and bonding studies [30,31,32,33,34]. RU is an extensively investigated conventional SARC, whereas TC has been introduced as a bioactive SARC with calcium-releasing potential, making these products appropriate comparators for evaluating the biological safety of newly developed BAG-containing formulations [33,34]. Although the viability in most groups slightly decreased at 72 h relative to that at 24 h, this trend may reflect the general time-dependent effects of prolonged eluate exposure rather than material-specific toxicity [35]. These findings are clinically relevant, particularly in situations involving thin residual dentin, where leachable acidic monomers or unreacted components from SARCs may diffuse through the dentinal tubules and potentially irritate the pulp [36]. Within the limitations of the eluate-based in vitro model, the present results suggest that the experimental BAG-containing SARCs did not exert pronounced cytotoxic effects on pulp-derived stem cells during the early exposure period, which supports their cytocompatibility as luting materials intended for direct contact with dentin.

The qRT-PCR analysis revealed that early odontogenic differentiation markers, including *RUNX2*, *ALP*, and *COL1A1*, did not exhibit consistent or dose-dependent expression patterns in the experimental groups (Figure 3a–c). Although sporadic upregulation was observed at some time points—such as increased *RUNX2* expression in the BG1 group on day 7; elevated *ALP* expression in the BG0, BG2, BG3, BG5, and RU groups on day 14; and increased *COL1A1* expression in the BG4 and BG5 groups on day 14—these changes were not uniform across time or BAG concentrations. This variability suggests that BAG incorporation did not have a robust or sustained influence on the early stages of odontogenic differentiation, which is primarily associated with lineage commitment and initial matrix synthesis [37].

In contrast, a more pronounced and systematic response was observed for the late-stage odontogenic markers. The expression of *DMP-1* and *DSPP* was significantly upregulated in the BAG-containing groups, particularly in BG2–BG5, across multiple time points (days 3, 7, and 14), indicating a sustained enhancement of late odontogenic differentiation (Figure 3d,e). Although the commercially available control cements (RU and TC) also exhibited increased *DMP-1* expression at certain time points, the BG groups showed more consistent responses over time. Notably, the relationship between BAG concentration and gene expression was not strictly linear, whereas *DSPP* expression tended to be more pronounced at higher BAG contents (BG3–BG5), suggesting a concentration-dependent tendency toward enhanced dentin-matrix-related maturation. These findings indicate that incorporating BAG particles into SARCs preferentially promoted the maturation and mineralization phases of odontogenic differentiation rather than early commitment, thereby supporting the role of BAG-containing SARCs in facilitating late-stage dentin matrix formation and mineral deposition [22,38,39].

Consistent with the qRT-PCR findings, IF staining performed on day 7 demonstrated distinct spatial expression patterns and markedly enhanced fluorescence intensities for *DMP-1* and *DSPP* in DPSCs cultured with BAG-containing SARCs. In particular, the BG groups—particularly BG2–BG5—exhibited significantly stronger fluorescence signals than the RU and TC groups, indicating that odontoblastic differentiation was upregulated at the protein level (Figure 4a,b). Although the OM group displayed moderate fluorescence intensity, consistent with its role as a positive differentiation control, the RU and TC groups showed comparable or lower expression levels. These observations suggest that the enhanced protein expression of *DMP-1* and *DSPP* was attributable to the incorporation of BAG within the experimental formulations rather than to baseline differentiation induced by the odontogenic medium alone.

Quantitative image analysis using ImageJ further substantiated these qualitative findings, revealing significantly greater fluorescence intensities in BG2–BG5 than in the OM group (*p* < 0.05), whereas no significant differences were observed in the RU and TC groups. The spatial distribution patterns of *DMP-1* and *DSPP* also differed, which is consistent with their distinct biological functions and intracellular processing pathways [40,41]. *DMP-1* staining revealed an elongated and fibrillar distribution along the cellular axis and perinuclear regions, corresponding to its established role in extracellular matrix organization and the regulation of mineral deposition. In contrast, *DSPP* staining revealed fine punctate granules localized within the cytoplasm, suggesting vesicular accumulation prior to secretion. This intracellular localization reflects the storage phase of *DSPP* before its release into the extracellular matrix, where it undergoes proteolytic cleavage into dentin sialoprotein and dentin phosphoprotein during the active phase of mineralization.

ARS staining revealed a time-dependent increase in mineralized nodule formation across all groups from days 14 to 28, indicating progressive extracellular matrix maturation. The OM group consistently yielded the highest O.D. values, validating its role as a positive control for robust mineralization. Formulations incorporating BAG demonstrated significantly more mineral deposition than the GM and conventional SARC groups (RU and TC), particularly on days 21 and 28. This time-dependent enhancement in the mineral deposition is consistent with previous studies demonstrating that BAG-derived ionic dissolution products promote late-stage odontogenic maturation, with mineralized nodule formation becoming more pronounced after approximately 2–3 weeks of culture, and robust ARS-positive calcification is typically observed around days 21 and 28 [22,38,42]. Although total mineralization in the BG groups did not reach the OM levels, these formulations outperformed the conventional SARCs during specific temporal windows, creating a microenvironment that was highly conducive to late-stage matrix mineralization. Notably, by day 28, the low-concentration groups (BG1 and BG2) had significantly higher O.D. values than the high-concentration groups (BG3–BG5). This indicates an optimal concentration window for BAG (approximately 1–2 wt%) rather than a strictly dose-dependent relationship.

The concentration-dependent mineralization pattern observed in the present study may be mechanistically explained by the coordinated biological effects of calcium and silicate ions released from the BAG particles. BAG is known to undergo surface reactions in aqueous environments, resulting in the sustained release of Ca^2+^ and soluble silicate species, both of which actively modulate cellular behavior. Calcium ions are crucial to regulating differentiation through calcium-sensing receptors and downstream signaling pathways, thereby influencing matrix maturation and mineral deposition [43]. Meanwhile, silicate ions have been reported to stimulate the expression of mineralization-related genes and promote extracellular matrix organization. Importantly, cellular responses to these ionic dissolution products are not strictly linear; rather, they are governed by ion-release kinetics and local concentration thresholds [19]. Consistent with the ARS staining results, excessive silicate ions in BAG have been reported to partially inhibit stem cell differentiation, suggesting that overly elevated ion levels may overwhelm cellular regulatory mechanisms and impede matrix maturation [44]. Moderate levels of calcium and silicate ions can enhance differentiation and apatite nucleation, whereas excessive ionic concentrations may disrupt intracellular homeostasis, alter the pH balance, or induce stress-related signaling, ultimately attenuating matrix maturation [45,46]. This biphasic pattern plausibly explains the superior mineralization observed in the BG1 and BG2 groups at day 28 compared with the higher-concentration groups. Rather than following a simple dose-dependent pattern, BAG incorporation appears to function within an optimal bioactive window, in which controlled ion availability creates a microenvironment conducive to late-stage odontogenic maturation and extracellular matrix mineralization [45].

Interestingly, although higher BAG concentrations (BG3–BG5) induced the most pronounced upregulation of *DSPP* mRNA expression, actual mineral deposition was most prominent in the lower-concentration groups (BG1 and BG2). This apparent discrepancy between transcriptomic profiles and phenotypic mineralization outcomes can be explained by the dose-dependent physicochemical dynamics of BAG dissolution [47]. On the one hand, excessive release of calcium and silicate ions from high BAG concentrations strongly triggers intracellular signaling pathways, driving high levels of initial gene transcription. However, this sustained high ionic burst may disrupt intracellular homeostasis or induce cellular stress, hindering post-transcriptional translation and secretion of extracellular matrix proteins [19]. Furthermore, the massive release of alkali ions from high-concentration BAG creates a hyperalkaline microenvironment. While mild alkalinity promotes biomineralization, an excessively high local pH (>9.0) downregulates ALP enzyme activity and physically impedes the precipitation of calcium phosphate crystals in pulp-derived cells [48]. Therefore, the lower concentrations of BAG (1–2 wt%) appear to provide the optimal balance, stimulating sufficient odontogenic gene expression without creating a hyperosmotic or excessively alkaline environment that suppresses late-stage matrix maturation.

Conversely, the TC group exhibited the lowest O.D. values throughout the study, despite its calcium silicate composition, which is typically associated with bioactivity and apatite formation. This diminished mineralization may have resulted from high local alkalinity, which can render the microenvironment suboptimal for sustained cellular differentiation. Furthermore, variations in the polymerization efficiency and the potential elution of residual monomers likely contributed to the attenuated cellular response in this group.

These results may be attributed, at least in part, to the ionic dissolution products released from the BAG particles, including calcium and silicate ions, which are known to modulate the local microenvironment and promote mineralization-favorable conditions. Previous studies demonstrated that BAG undergoes surface reactions in aqueous environments, leading to the sustained release of biologically active ions that can stimulate cellular activity and apatite formation [17,21]. Moreover, BAG-containing materials can generate a localized alkaline microenvironment owing to ion exchange and hydroxycarbonate apatite formation. Such pH modulation has been reported to influence cellular behavior, including the proliferation and differentiation of mineralizing cells [49]. In the context of SARCs, which intrinsically possess acidic functional monomers, the incorporation of BAG may partially counteract the localized acidity and create a more favorable milieu for DPSC differentiation and extracellular matrix mineralization. Therefore, the increases in both the expression of late odontogenic markers and mineral deposition in the present study may reflect the combined effects of controlled ion release, microenvironmental alkalization, and the bioactive properties of BAG.

Although no marked differences in cytocompatibility were detected among the investigated SARCs in the MTT assay, the BG groups elicited distinctly enhanced late-stage odontogenic differentiation and mineralization responses. This apparent dissociation between early viability and later functional outcomes is biologically plausible, as resin-based eluates may not necessarily produce overt cytotoxicity within the early exposure window yet can still modulate lineage-associated maturation and matrix mineralization through microenvironmental cues and ionic signaling [50,51]. In this context, BAG-derived ionic dissolution products have been shown to promote odontogenic differentiation and mineralized matrix formation in pulp-derived cells, supporting the interpretation that incorporating BAG contributes to a more mineralization-favorable milieu during the later differentiation phase [19,22,38].

Conversely, the TC group exhibited the lowest mineral deposition in the ARS analyses throughout the study, despite its calcium silicate composition [34]. Although the calcium-releasing characteristics of TC and the resulting alkaline pH can be clinically advantageous for certain applications, excessively high local alkalinity may render the microenvironment suboptimal for sustained cellular differentiation and matrix maturation [48]. Furthermore, the biological performance of TC may have been further compromised by variations in the polymerization efficiency and the subsequent elution of residual monomers [13,16]. Because unreacted resin monomers are known to interfere with odontogenic differentiation in pulp-derived cells, these combined physicochemical factors likely explain the attenuated mineralization in the TC group [50,51]. Collectively, these findings highlight how incorporating BAG into experimental SARCs provided a more conducive milieu for late-stage odontogenic maturation and mineral deposition.

Despite these encouraging results, several limitations must be acknowledged. First, this study employed an eluate-based in vitro model, which cannot fully replicate the dynamic clinical environment of the dentin–pulp complex. In vivo, factors such as the remaining dentin thickness, dentinal tubule permeability, and outward pulpal pressure actively regulate the diffusion of material components and subsequent pulpal exposure. Additionally, the reliance on a 2D single-cell culture system does not capture the complex intercellular and matrix interactions inherent to native pulp tissue. Future investigations should employ organoid models or dentin barrier tests to better simulate the physiological barriers and structural complexity of teeth.

Furthermore, although phenotypic outcomes were evident, the specific mechanisms underlying enhanced differentiation and mineralization in the BAG-modified groups were not directly quantified. In particular, the lack of data on ion release kinetics and the physical properties limits a comprehensive understanding of the interaction between SARCs and DPSCs. To address this, further studies will be conducted to thoroughly evaluate these exact physiochemical parameters. Finally, long-term in vivo animal studies are warranted to confirm whether the optimal BAG concentration window (1–2 wt%) identified in vitro effectively translates into improved pulpal healing and robust tertiary dentin formation under clinically relevant conditions.

## 5. Conclusions

Within the limitations of this in vitro study, the incorporation of BAG into SARCs did not compromise the short-term viability of DPSCs. Notably, BAG-modified formulations more significantly enhanced late-stage odontogenic differentiation and mineralization compared with conventional SARCs. This biological enhancement was most pronounced at moderate BAG concentrations, indicating an optimal bioactive window rather than a strict dose-dependent relationship. These findings suggest that optimizing the BAG content can substantially improve the biological performance of SARCs by making the microenvironment more conducive to dentin matrix maturation. Further in vivo studies should validate the long-term pulpal healing capacity and the clinical relevance of these novel materials.

## Figures and Tables

**Table 1:** Compositions of the materials used in the study according to the manufacturers.

Material	Main Composition		Manufacturer
BAG-containing SARCs (BG0 to BG5)	Base paste Methacrylate monomers, TEGDMA, BAG (0–5 wt%), silanized silica, Barium glass, additives	Catalyst paste Methacrylate monomers, TEGDMA, BAG (0–5 wt%), silanized silica, Barium glass, additives	Mediclus, Cheongju, Republic of Korea
RelyX U200 (RU)	Base paste Fiberglass, phosphoric acid, methacrylate esters, TEGDMA, silane-treated silica, sodium persulfate	Catalyst paste Fiberglass, substitute dimethacrylate, silane-treated silica, P-toluene sulfonate sodium, calcium hydroxide	3M ESPE, St. Paul, MN, USA
TheraCem (TC)	Base paste Calcium base filler, glass fillers, bisphenol-A-diglycidylmethacrylate, dimethacrylates, 2-hydroxyethyl methacrylate, ytterbium fluoride, initiator, amorphous silica	Catalyst paste Glass fillers, 10-MDP, amorphous silica	Bisco, Schaumburg, IL, USA

Abbreviations: TEGDMA, triethylene glycol dimethacrylate; BAG, bioactive glass; 10-MDP, 10-methacryloyloxydecyl dihydrogen phosphate. The precise properties of the incorporated BAG, including its exact particle size, morphological features, and elemental composition, are classified as proprietary information by the manufacturer (Mediclus) and were not disclosed in this study.

**Table 2:** Growth medium and odontogenic medium compositions.

Growth Medium	Odontogenic Medium
α-Minimum essential medium 10% Fetal bovine serum Penicillin/Streptomycin (100 U/mL) 2 mM L-Glutamine	α-Minimum essential medium 10% Fetal bovine serum Gentamycin reagent solution (50 μg/mL) 100 μM L-ascorbic acid 10 nM Dexamethasone 10 mM β-Glycerol phosphate 1.8 mM Monopotassium phosphate

**Table 3:** Primer sequences used for qRT-PCR.

Gene	Gene Name	Forward Sequence	Reverse Sequence
*RUNX2*	*Runt-related transcription factor 2*	5′-GGTTAATCTCCGCAGGTCACT-3′	5′-CACTGTGCTGAAGAGGCTGTT-3′
*ALP*	*Alkaline phosphatase*	5′-GACAAGAAGCCCTTCACTGC-3′	5′-AGACTGCGCCTGGTAGTTGT-3′
*COL1A1*	*Collagen type I alpha 1*	5′-CCTGGAAAGAATGGAGATGATG-3′	5′-ATCCAAACCACTGAAACCTCTG-3′
*DMP-1*	*Dentin matrix protein 1*	5′-TCCACAGTACCGGATTCTCTCT-3′	5′-TCTATGTTAGCACCTTGTCTCCA-3′
*DSPP*	*Dentin sialophospho protein*	5′-ATATTGAGGGCTGGAATGGGGA-3′	5′-TTTGTGGCTCCAGCATTGTCA-3′
*ß-actin*	*Beta-actin*	5′-GGCACCCAGCACAATGAAG-3′	5′-TGCGGTGGACGATGGAGG-3′

## Data Availability

The raw data supporting the conclusions of this article will be made available by the authors on request.

## Abbreviations

The following abbreviations are used in this manuscript:

ALP	alkaline phosphatase
ANOVA	one-way analysis of variance
ARS	Alizarin Red S
BAG	bioactive glass
COL1A1	collagen type I alpha 1
DAPI	4′,6-diamidino-2-phenylindole
DMP-1	dentin matrix protein 1
DPSC	dental pulp stem cell
DSPP	dentin sialophosphoprotein
GM	growth medium
HEMA	2-hydroxyethyl methacrylate
IF	immunofluorescence
MTT	methylthiazol tetrazolium
O.D.	optical density
PBS	odontogenic medium
qRT-PCR	phosphate-buffered saline
RU	RelyX U200
RUNX2	runt-related transcription factor 2
SARC	self-adhesive resin cement
TEGDMA	triethylene glycol dimethacrylate
TC	TheraCem
10-MDP	10-methacryloyloxydecyl dihydrogen phosphate
